# Violence Against LGB+ people in Brazil: analysis of the 2019 National Survey of Health

**DOI:** 10.1590/1980-549720230005.supl.1

**Published:** 2023-04-21

**Authors:** Nádia Machado de Vasconcelos, Francielle Thalita Almeida Alves, Gisele Nepomuceno de Andrade, Isabella Vitral Pinto, Adauto Martins Soares, Cimar Azeredo Pereira, Deborah Carvalho Malta

**Affiliations:** I Universidade Federal de Minas Gerais, School of Medicine, Graduate Program in Public Health – Belo Horizonte (MG), Brazil.; II Universidade Federal de Minas Gerais, School of Medicine – Belo Horizonte MG, Brazil.; III Universidade Federal de Minas Gerais, School of Medicine, Graduate Program in Nursing – Belo Horizonte (MG), Brazil.; IV Ministry of Health, Department of Noncommunicable Diseases – Brasília (DF), Brazil.; V Instituto Brasileiro de Geografia e Estatística – Rio de Janeiro (RJ), Brazil.; VI Universidade Federal de Minas Gerais, School of Medicine, Department of Maternal-Child and Public Health Nursing – Belo Horizonte (MG), Brazil.

**Keywords:** Sexual and gender minorities, Sexism, Gender-based violence, Health surveys

## Abstract

**Objective:**

To analyze the association between self-reported sexual orientation and violence in the Brazilian population.

**Methods:**

This cross-sectional epidemiological study used the 2019 National Survey of Health database. Total violence and its subtypes (psychological, physical, and sexual) were analyzed in the previous 12 months. Prevalence and odds ratio adjusted for age group were estimated, with their respective 95% confidence intervals, according to the self-reported sexual orientation of the Brazilian population aged 18 years and older. Statistical significance was set at 5%.

**Results:**

Most of the Brazilian population self-identified as heterosexual (94.75%) and 1.89% as LGB+. This percentage was lower than that of respondents who refused to answer the question (2.28%). The prevalence of violence in the general population of Brazil was 18.27%, and the most common subtype was psychological violence (17.36%). The LGB+ population was more than twice as likely to experience any type of violence. LGB+ women had the highest prevalence in all violence subtypes, and heterosexual men had the lowest. LGB+ women were over three times more likely to experience physical violence compared to heterosexual ones. Meanwhile, the probability of LGB+ men experiencing sexual violence was almost eight times higher than in heterosexual men.

**Conclusion:**

The prevalence of violence against the LGB+ population was high in the country. Public policies aimed at this population are necessary to fight discrimination against sexual diversity and ensure the rights of non-heterosexual people.

## INTRODUCTION

Cis-heteronormativity can be understood as a system of power relations that assumes the existence of only two opposing genders (man and woman), which always coincide with their bodies (male and female biological sex) and will always be mutually attracted by their opposite^1^. However, sexual orientation and gender identity can have multiple characteristics and affective-sexual behaviors that differ from this normativity. People with this so-called dissenting self-identification comprise the LGBTQIA+ population, an umbrella term encompassing, but not limited to, lesbian, gay, bisexual, transgender, queer, intersex, and asexual people, and others^2^. The population of lesbians, gays, bisexuals, and other sexual minorities (LGB+), the object of this study, consists of individuals whose behaviors, desires, and/or emotional-affective-sexual identity differ from those defined for cisgender heterosexuals^3,4^. For contradicting the expected established standard, this group faces discrimination, vulnerabilities, and invisibility, experiencing prejudice against sexual diversity^5^.

One of the consequences of this discrimination is their greater susceptibility to violence. Violence is a public health problem, and its confrontation is part of the 2030 Agenda for Sustainable Development Goals^6^. People exposed to violence may present unfavorable health outcomes, not only psychological but also physical and sexual. Among the main consequences of violent acts, depression, post-traumatic stress disorder, fractures, and head trauma stand out, as well as sexually transmitted infections and unintended pregnancies^7^.

A report by the Bahia Gay Group (*Grupo Gay da Bahia* — GGB), a non-governmental organization (NGO) that since 1980 collects data on violence against sexual and gender minorities in Brazil, revealed that 165 gays and lesbians were killed in the country as a result of discrimination against their sexual orientation in 2021^8^. In addition, a study based on data from the Notifiable Diseases Information System (*Sistema de Informação de Agravos de Notificação* — SINAN) found 13,129 reports against homosexuals and bisexuals between 2015 and 2017, a number that may be underestimated due to possible undernotification^9^. Moreover, the scarcity of information about this group in Brazil stands out, which prevents knowing its profile and identifying its needs, therefore affecting the formulation of public policies aimed at this social minority^10^.

In order to advance in this scenario, the 2019 National Survey of Health (*Pesquisa Nacional de Saúde* — PNS) included an investigation on the sexual orientation of adult Brazilians for the first time in a national epidemiological survey of the Brazilian Institute of Geography and Statistics (*Instituto Brasileiro de Geografia e Estatística* — IBGE)^11^. The addition of this theme raises multiple possibilities of analysis regarding health, as well as risk and protective factors, for the LGB+ population, including violence.

The study of violence is very relevant in the context of the LGB+ population, as it allows understanding how complex is the vulnerability to which this group is exposed. Thus, knowing the characteristics of this problem favors the implementation and strengthening of public policies to cope with discrimination against this population.

From this perspective, the present study aimed to analyze, for the first time, the association between self-reported sexual orientation and violence in the Brazilian population.

## METHOD

### Data design and source

This is a cross-sectional epidemiological study, with an analytical and quantitative approach, based on data from the 2019 PNS.

The 2019 PNS assessed individuals aged 15 years or older living in Brazil. The sample had a three-stage cluster design, namely: 1. census tracts or set of tracts; 2. households; and 3. residents. A total of 108,525 households were selected for the interview, and the final sample comprised 90,846 interviews conducted, with a response rate of 96.5%. A specific publication provides further details on the 2019 PNS methodology^12^. The present study selected individuals aged 18 years or older who answered the Violence and Sexual Activity modules.

### Variables

The outcome variable — violence — was constructed using questions from the Violence module (V). Violence was considered present when the interviewee answered “yes” to any option of questions concerning psychological, physical, and sexual violence (questionnaire details can be found at: https://www.pns.icict.fiocruz.br/wp-content/uploads/2021/02/Questionario-PNS-2019.pdf).

Sexual orientation — explanatory variable — was analyzed based on the answer to the question “What is your sexual orientation?” in the Sexual Activity module (Y) and categorized into heterosexual; LGB+ (homosexual, bisexual, and other orientations); does not know; and refused to answer.

Descriptive sociodemographic variables were also selected: gender (male and female); age group (18 to 29 years; 30 to 39 years; 40 to 59 years; 60 years or older); schooling (illiterate and incomplete elementary school; complete elementary school and incomplete high school; complete high school and incomplete higher education; and complete higher education); ethnicity/skin color (white, black [including biracial], others [Asian and indigenous]); region (North, Northeast, Southeast, South, and Midwest); income (up to one minimum wage (MW); more than one and up to three MWs; more than three MWs); place of residence (urban or rural); and marital status (single; married; widower; divorced or legally separated).

### Data analysis

In the descriptive analysis, the distribution of sexual orientation and their respective 95% confidence intervals (95%CI) were calculated, as well as the distribution according to sociodemographic characteristics. The prevalence of total violence and its subtypes — total and stratified by self-reported gender in the PNS — were also calculated.

A multivariate analysis between the outcome variables (total violence, psychological violence, physical violence, and sexual violence) and each category of the explanatory variable (sexual orientation) was performed to evaluate the association of violence with sexual orientation. Previous studies^13,14^ have shown that age group is an important factor associated with violence and, therefore, can be considered a confounding factor. Thus, the option adopted was to estimate the odds ratio adjusted (ORad) for age group and the respective 95%CI using logistic regression, an appropriate technique validated for cross-sectional studies^15^. The final model adopted a 5% significance level.

Thanks to the complex sampling design and the distinct selection probabilities, the PNS analysis employed sample weights, and the final weight used is the product of inverted expressions of the selection likelihood in each sample stage. It should be noted that final weight encompasses the correction of non-responses and adjustments for population totals^12^.

Analyses of the present study were performed in the Software for Statistics and Data Science (Stata), version 14.0, in its survey module, which considers the effects of the sampling plan.

### Ethical aspects

All participants gave their consent at the time of the interview. The National Research Ethics Committee/National Health Council approved the PNS project under opinion no. 3,529,376, issued on August 23, 2019.

## RESULTS

A total of 88,531 individuals aged 18 years or older who answered the Violence and Sexual Activity modules of PNS were analyzed. Most of the interviewees self-identified as heterosexuals (94.75%; 95%CI 94.46–95.03) and 1.89% (95%CI 1.73–2.07) as LGB+. This percentage was lower than that of respondents who refused to answer the question (2.28%; 95%CI 2.08–2.48) (Table 1).

**Table 1:** Distribution of self-reported sexual orientation of Brazilians aged 18 years or older (n=88,531). National Survey of Health, 2019.

Sexual orientation	Distribution (95%CI)		
	Total	Men	Women
**Heterosexual**	**94.75 (94.46–95.03)**	**94.70 (94.25–95.12)**	**94.80 (94.43–95.16)**
**LGB+**	**1.89 (1.73–2.07)**	**1.93 (1.69–2.20)**	**1.86 (1.64–2.11)**
Bisexual	0.68 (0.58–0.80)	0.50 (0.38–0.66)	0.84 (0.69–1.03)
Homosexual	1.15 (1.03–1.29)	1.40 (1.21–1.62)	0.93 (0.79–1.11)
Other orientations	0.06 (0.03–0.08)	0.03 (0.01–0.05)	0.09 (0.05–0.14)
**Does not know**	**1.08 (0.95–1.23)**	**1.08 (0.89–1.32)**	**1.08 (0.92–1.26)**
**Refused to answer**	**2.28 (2.08–2.48)**	**2.29 (2.00–2.62)**	**2.26 (2.02–2.52)**
**Total**	100.00	100.00	100.00

95%CI: 95% confidence interval.

Regarding sociodemographic characteristics, people aged 18 to 29 years had the highest self-reported LGB+ orientation (4.91%; 95%CI 4.31–5.59) and were the ones who most refused to answer (3.19%; 95%CI 2.72–3.75) among age groups. The percentage of LGB+ self-identification was also higher in people with household income above three MWs (3.30%; 95%CI 2.73–3.99), as well as those living in urban areas (2.05%; 95%CI 1.87–2.26). As for marital status, single people had a higher percentage of LGB+ self-identification (3.81%; 95%CI 3.45–4.20), as well as refusal to answer (2.76%; 95%CI 2.47–3.09) (Supplementary Material — Table 1).

**Figure 1:**
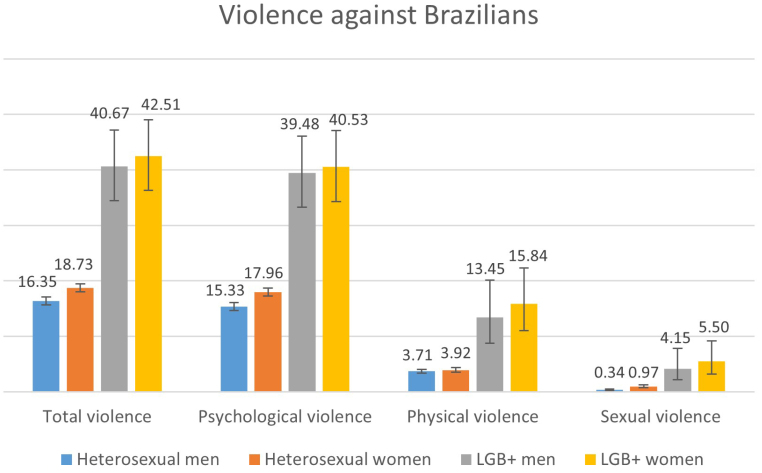
Prevalence, with a 95% confidence interval, of violence and its subtypes stratified by gender and sexual orientation. National Survey of Health, 2019.

In 2019, the prevalence of total violence in Brazil was 18.27% (95%CI 17.74–18.81). In all violence subtypes, the highest prevalence rates were found among LGB+ women [(psychological: 40.53%; 95%CI 34.29–47.09); (physical: 15.84%; 95%CI 10.99–22.28); (sexual: 5.50%; 95%CI 3.23–9.20)], while heterosexual men had the lowest [(psychological: 15.33%; 95%CI 14.62–16.08); (physical: 3.71%; 95%CI 3.39–4.06); (sexual: 0.34%; 95%CI 0.25–0.46)] Figure 1).

In relation to total violence, LGB+ people were 2.52 (p<0.001) more likely to experience violence than heterosexuals; this probability was 2.69 (p<0.001) higher in men who self-identified as LGB+ than in heterosexual ones, while LGB+ women had 2.40 (p<0.001) times higher odds of experiencing violence than heterosexual ones. Similar percentages were found for psychological violence (Table 2).

**Table 2:** Prevalence and adjusted odds ratio, with a 95% confidence interval, of total violence and its subtypes experienced by Brazilians aged 18 years or older, according to sexual orientation and stratified by gender. National Survey of Health, 2019.

		Prevalence and adjusted odds ratio (95% confidence interval)					
		Total		Male		Female	
		Prevalence (95%CI)	Odds Ratio* (95%CI)	Prevalence (95%CI)	Odds Ratio* (95%CI)	Prevalence (95%CI)	Odds Ratio* (95%CI)
**Total violence**							
**Total**		18.27 (17.74–18.81)	–	17.01 (16.28–17.77)	–	19.38 (18.70–20.08)	–
**Sexual orientation**							
	Heterosexual	17.61 (17.08–18.16)	1.00 (–)	16.35 (15.62–17.11)	1.00 (–)	18.73 (18.04–19.43)	1.00 (–)
	LGB+	41.63 (37.14–46.27)	**2.52** **(2.08–3.07)**	40.67 (34.46–47.19)	**2.69** **(2.05–3.53)**	42.51 (36.26–49.01)	**2.40** **(1.83–3.14)**
	Does not know	29.27 (24.10–35.03)	**1.64** **(1.27–2.13)**	25.46 (18.23–34.37)	1.42 (0.93–2.16)	32.62 (25.80–40.26)	**1.84** **(1.33–2.54)**
	Refused to answer	20.96 (17.60–24.77)	1.16 (0.94–1.44)	20.44 (15.21–26.91)	1.17 (0.82–1.67)	21.43 (17.42–26.07)	1.16 (0.89–1.51)
**Psychological violence**							
**Total**		17.36 (16.84–17.90)	–	15.98 (15.26–16.72)	–	18.58 (17.91–19.27)	–
**Sexual orientation**							
	Heterosexual	16.73 (16.21–17.27)	1.00 (–)	15.33 (14.62–16.08)	1.00 (–)	17.96 (17.29–18.65)	1.00 (–)
	LGB+	40.03 (35.53–44.71)	**2.54** **(2.08–3.10)**	39.48 (33.26–46.06)	**2.79** **(2.12–3.67)**	40.53 (34.29–47.09)	**2.35** **(1.78–3.09)**
	Does not know	26.64 (21.56–32.42)	**1.54** **(1.17–2.03)**	22.41 (15.29–31.60)	1.31 (0.83–2.07)	30.38 (23.56–38.19)	**1.75** **(1.24–2.46)**
	Refused to answer	20.42 (17.08–24.21)	1.20 (0.97–1.50)	19.70 (14.53–26.14)	1.22 (0.85–1.74)	21.06 (17.06–25.70)	1.19 (0.91–1.56)
**Physical violence**							
**Total**		4.15 (3.89–4.42)	–	4.05 (3.71–4.41)	–	4.24 (3.84–4.67)	–
**Sexual orientation**							
	Heterosexual	3.82 (3.57–4.09)	1.00 (–)	3.71 (3.39–4.06)	1.00 (–)	3.92 (3.54–4.34)	1.00 (–)
	LGB+	14.70 (11.16–19.12)	**3.00** **(2.19–4.12)**	13.45 (8.74–20.13)	**2.84** **(1.74–4.65)**	15.84 (10.99–22.28)	**3.18** **(2.09–4.83)**
	Does not know	12.35 (8.33–17.92)	**2.84** **(1.84–4.38)**	13.93 (8.01–23.13)	**3.18** **(1.72–5.88)**	10.95 (6.21–18.59)	**2.52** **(1.36–4.67)**
	Refused to answer	4.94 (3.52–6.88)	1.19 (0.82–1.71)	5.37 (3.32–8.59)	1.25 (0.74–2.10)	4.55 (2.81–7.26)	1.13 (0.68–1.88)
**Sexual violence**							
**Total**		0.76 (0.65–0.90)	–	0.45 (0.35–0.57)	–	1.05 (0.85–1.28)	–
**Sexual orientation**							
	Heterosexual	0.68 (0.56–0.82)	1.00 (–)	0.34 (0.25–0.46)	1.00 (–)	0.97 (0.78–1.22)	1.00 (–)
	LGB+	4.86 (3.21–7.28)	**4.98** **(3.10–7.99)**	4.15 (2.15–7.86)	**7.76** **(3.38–17.81)**	5.50 (3.23–9.20)	**3.95** **(2.21–7.05)**
	Does not know	1.71 (0.94–3.11)	**1.96** **(1.03–3.72)**	2.15 (0.92–4.92)	**4.29** **(1.68–10.94)**	1.33 (0.60–2.90)	1.11 (0.48–2.57)
	Refused to answer	0.56 (0.31–1.02)	0.73 (0.39–1.36)	0.79 (0.36–1.72)	1.87 (0.79–4.42)	0.35 (0.15–0.85)	**0.34** **(0.13–0.85)**

Note:

* odds ratio adjusted for age group; bold values — p<0.05; 95%CI: 95% confidence interval.

Regarding physical violence, the probability was 2.84 (p<0.001) higher in LGB+ men than in heterosexual males, while for LGB+ women, this number was 3.18 (p<0.001) higher compared to heterosexual ones (Table 2).

Finally, the odds of an LGB+ person experiencing sexual violence were 4.98 (p<0.001) higher than in heterosexual people. LGB+ men were 7.76 (p<0.001) more likely to experience this subtype of violence than heterosexual ones, while in LGB+ women, this probability was 3.95 (p<0.001) higher than in heterosexual females (Table 2).

## DISCUSSION

This study analyzed data from the 2019 PNS and showed that approximately 2% of the Brazilian population self-identifies as LGB+, a percentage lower than those who refused to answer the question. About half of the LGB+ individuals reported having experienced some kind of violence in the previous 12 months, and they had around twice the chances of experiencing any type of violence compared to people who self-identified as heterosexuals. LGB+ women were the most common victims, while heterosexual men had the lowest rates. The odds ratio is higher for sexual violence — LGB+ people were almost five times more likely to be victims of this violence subtype.

The percentage of the population aged 18 years and older who self-identified as LGB+ in this study was below that found by a DataFolha survey conducted in 2018, which reached the rate of 4.42% of the Brazilian population^16^. The percentage of people who refused to answer the question about sexual orientation exceeded that of individuals who self-identified as LGB+. This fact shows that the issue of sexual diversity is stigmatizing in Brazil. The LGB+ population has historically experienced prejudice and discrimination of religious, moral, and even health care^17^ nature. Therefore, people often try to hide their dissenting orientation and refusing to answer the question can be a way to protect themselves^10^.

In addition, the percentage of people who did not know how to answer the question was similar to that of people who self-identified as LGB+. Since the topic of sexual orientation was addressed in a single question, some interviewees might have had difficulty understanding the question. One way to mitigate this would be to include better-known terms, such as lesbian and gay^10^.

This study showed that LGB+ people have more than twice the odds of experiencing violence in all subtypes. Discrimination against sexual diversity is a gender-based violence. Gender can be understood as a social construction in which biological sex dictates the role a person should play^18^. This structure also includes heteronormativity, which demands a standardized lifestyle from people, with postures and choices consistent with what has been determined to be right for men and women^19^. Thus, this social construction not only imposes a way to act and behave publicly but also establishes that each person must have affective and sexual relationships with the opposite gender^5^. Any deviation from this pattern is then a reason for prejudice and justification for violent acts^17^.

Our study revealed that women who self-identified as LGB+ had the highest prevalence rates of violence in all subtypes, while self-reported heterosexual men presented the lowest. This finding shows that the experience of violence increases with the accumulation of social vulnerabilities of these people. Lesbian and bisexual women face double discrimination: sexism and prejudice against sexual diversity. Our society devalues women due to the supposed male supremacy, in addition to disqualifying LGB+ people based on cis-heteronormativity^20^. Consequently, lesbians and bisexual women are more vulnerable to violence. A systematic review that analyzed articles with data from 50 countries found a prevalence of up to 25% of physical violence and 13.2% of sexual violence in this population^21^.

People who self-identified as LGB+ were three times more likely to experience physical violence than heterosexuals, and these odds were higher among women than among men. This finding is compatible with another study that analyzed the reports of violence between 2015 and 2017 and found that, among adults, the main subtype reported is physical violence, with lesbians as the main victims^9^. Once again, this information reinforces the intersectionality of vulnerabilities.

The present study also found that LGB+ people were almost five times more likely to experience sexual violence than heterosexuals, with this violence subtype showing the greatest difference between heterosexuals and LGB+. Among many issues involving this situation is the occurrence of “corrective rape”, in which non-heterosexual people are subjected to abuses from an aggressor whose intention is to control the victim's social and/or sexual behavior^22^.

Furthermore, the study indicated that LGB+ men were approximately eight times more likely to experience such violence than heterosexual men, while these odds were close to four times higher among LGB+ women compared to heterosexual ones. The DataFolha study found similar information: the proportion of gay men who reported sexual violence was more than ten times higher than that of heterosexual ones, while lesbians reported approximately 1.5 times more sexual violence than heterosexual women^16^. It should be noted that heterosexual men are the ones who least experienced this type of violence and, due to the low prevalence of this violence and the small sample, these percentages might not be accurate. However, the fact that men may commit violent acts against their partners due to internalized homophobia^23^ and the social creation of conflict resolution through violence^24^ should also be considered. Further studies are necessary to better understand this result.

Because they constitute a vulnerable population, LGB+ people demand public policies to guarantee their human and health rights. In Brazil, the current constitution does not specifically address this population, a defeat for the movement organized for LGBTQIA+ rights in the country^25^. Nonetheless, some achievements have been made over the last decades, such as the guarantee of the right to civil marriage between same-sex people in 2013 and the classification of discriminatory acts as a crime equivalent to racism in 2019. In the health area, the National Policy for the Comprehensive Health of Lesbians, Gays, Bisexuals, Transvestites, and Transgender people was developed in 2013^26^, and, more recently, the possibility of homosexuals and bisexuals donating blood was recognized in 2020^27^.

In the past few years, though, Brazil has experienced a period of socio-political crisis, and the advancement of conservatism has limited LGBTQIA+ achievements and imposed setbacks on this population. Since 2018, organizations aimed at protecting the LGB+ population have been terminated, such as the Secretariat for Continuing Education, Literacy, Diversity, and Inclusion and the National Council for Combating LGBT Discrimination, in addition to government representatives systematically targeting this population with discriminatory statements and hate speech, including with legal representation to overturn the decision that equated homophobia with racism^28^. Thus, public policies for the protection of the LGB+ population still have much to advance, with the construction of a legal framework to defend the rights of this population, budget forecasting for plans and programs, and greater political representativeness of LGB+ people^25^.

The study data refer to the last year before the COVID-19 pandemic declared by the World Health Organization in 2020. In the pandemic context, social distancing, increased stress, and possible exposure to disrespectful family members aggravated the risk of violence for the LGB+ population^29^. Moreover, the economic insecurity during the pandemic crisis, with the growth of unemployment and poverty in the country, intensified the fragilities already faced by these people^30^. Therefore, the 2019 PNS data can be used as a baseline for further analyses of the prevalence of violence against LGB+ people during and after the global health crisis.

The 2019 PNS was the first Brazilian population-based epidemiological household survey to have a question related to sexual orientation, which can contribute to studies of this population and produce scientific evidence to help the construction of effective public policies to ensure the rights of these people. Nevertheless, the questionnaire needs to be expanded, including questions related to gender identity, in order to give visibility to all vulnerabilities to which non-cis-heteronormative people are exposed.

Among the limitations of this study is the fact that data on sexual orientation are considered experimental, and IBGE points out that analyses should be performed with caution. In addition, the sample does not include homeless populations, nursing homes, *quilombos*, and villages, and the survey does not have all violence subtypes, leaving moral and patrimonial violence out, for example. Additionally, the prevalence rates calculated herein might be underestimated, given the stigma that violence and non-heterosexual orientation have in society, leading people not to report these facts. To try to minimize this last limitation, the interviewee's privacy could be guaranteed when they answer modules related to such questions, besides allowing them to respond directly on the device, reducing possible embarrassment.

In conclusion, the 2019 PNS data revealed a high prevalence of violence against LGB+ people in Brazil, highlighting the great vulnerability of this population to such acts. Thus, this study provides scientific evidence of the need for intersectoral coordination involving health, education, justice, and public safety, among others, to combat this violence, with the formulation of public policies that fully protect the lives and rights of LGB+ people.

## References

[1] Rosa EBPR (2020). Cisheteronormatividade como instituição total. Cad PET-Filosofia.

[2] Cooper KM, Brownell SE (2016). Coming out in class: Challenges and benefits of active learning in a biology classroom for LGBTQIA students. CBE Life Sci Educ.

[3] Sociedade Brasileira de Medicina de Família e Comunidade (2020). Cartilha Mitos e Verdades sobre saúde da população LGBTIA+. 1ᵃ ed [Internet].

[4] Reis T (2018). Manual de Comunicação LGBTI+ [Internet].

[5] Costa ÂB, Nardi HC (2015). Homophobia and prejudice against sexual diversity: Conceptual debate. Temas Psicol.

[6] United Nations (2015). Transforming our world: the 2030 agenda for sustainable development [Internet].

[7] Krug EG, Dahlber LL, Mercy JA, Zwi AB, Lozano R (2002). World report on violence and health [Internet].

[8] Oliveira JMD, Mott L (2022). Mortes violentas de LGBT+ no Brasil: relatório 2021 [Internet].

[9] Pinto IV, Andrade SSA, Rodrigues LL, Santos MAS, Marinho MMA, Benício LA (2020). Perfil das notificações de violências em lésbicas, gays, bissexuais, travestis e transexuais registradas no Sistema de Informação de Agravos de Notificação, Brasil, 2015 a 2017. Rev Bras Epidemiol.

[10] Carvalho AA, Barreto RCV (2021). The invisibility of the LGBTQIA+ people in the databases: New possibilities in the 2019 national health research?. Ciênc Saúde Coletiva.

[11] Instituto Brasileiro de Geografia e Estatística (2022). Orientação sexual autoidentificada da população adulta [Internet].

[12] Stopa SR, Szwarcwald CL, Oliveira MM de, Gouvea ECDP, Vieira MLFP, Freitas MPS (2020). Pesquisa Nacional de Saúde 2019: histórico, métodos e perspectivas. Epidemiol Serv Saúde.

[13] Instituto Brasileiro de Geografia e Estatística (2020). Pesquisa Nacional de Saúde 2019: acidentes, violências, doenças transmissíveis, atividade sexual, características do trabalho e apoio social [Internet].

[14] Instituto Brasileiro de Geografia e Estatística (2015). Acesso e utilização dos serviços de saúde, acidentes e violências: Brasil, grandes regiões e unidades da federação.

[15] Coutinho LMS, Scazufca M, Menezes PR (2008). Methods for estimating prevalence ratios in cross-sectional studies. Rev Saude Publica.

[16] Spizzirri G, Eufrásio RÁ, Abdo CHN, Lima MCP (2022). Proportion of ALGBT adult Brazilians, sociodemographic characteristics, and self - reported violence. Sci Rep.

[17] Peixoto VB (2018). Violência contra LGBTs no Brasil: premissas históricas da violação no Brasil. Rev Periódicus.

[18] Scott J (2017). Gênero: uma categoria útil de análise histórica. Educ Real [Internet].

[19] Colling L, Nogueira G, Rodrigues A, Dallapicula C, Ferreira SR da S (2015). Transposições: lugares e fronteiras em sexualidade e educação.

[20] Santana PF, Rasera EF (2018). Heterossexismo e a (in)existência lésbica. Rev Psicol UNESP [Internet].

[21] Blondeel K, Vasconcelos S, García-Moreno C, Stephenson R, Temmerman M, Toskin I (2018). Violence motivated by perception of sexual orientation and gender identity: a systematic review. Bull World Health Organ [Internet].

[22] Costa LSU (2021). A prática delitiva do estupro corretivo e a heteronormatividade compulsória: um estudo acerca da correlação entre crime e patriarcado. Rev Dir Sex.

[23] Antunes PPS (2018). Anais do XI Seminário Internacional Fazendo Gênero.

[24] Santos AMR, Caridade SMM (2017). Violência nas relações íntimas entre parceiros do mesmo sexo: estudo de prevalência. Temas Psicol.

[25] Sousa CAA, Mendes DC (2021). Políticas públicas para a população LGBT: uma revisão de estudos sobre o tema. Cad EBAPEBR.

[26] Brasil (2013). Política nacional de saúde integral de lésbicas, gays, bissexuais, travestis e transsexuais [Internet].

[27] Oliveira BA, Irineu BA, Irineu BA, Lopes MA, Rocon PC, Silva MA, Nascimento MAN, Duarte MJ (2021). Diversidade sexual, étnico-racial e de gênero: saberes plurais e resistências [Internet].

[28] Irineu BA, Lacerda BA, Irineu BA, Nascimento MAN, Lopes MA, Rocon PC, Jesus DM, Passamani GR (2020). Diversidade sexual, étnico-racial e de gênero: temas emergentes.

[29] United Nations (2020). La violencia y la discriminación por motivos de orientación sexual o identidad de género y la identidad de género durante la pandemia de enfermedad coronavirus (COVID-19) [Internet].

[30] Bordiano G, Liberal SP, Lovisi GM, Abelha L (2021). COVID-19, social vulnerability and mental health of LGBTQIA+ populations. Cad Saude Publica.

