# Supplementary material for: Violence Against LGB+ people in Brazil: analysis of the 2019 National Survey of Health
Source: Rev Bras Epidemiol. 2023 Apr 21;26(Suppl 1):e230005. doi: 10.1590/1980-549720230005.supl.1 (PMC10176725; doi:10.1590/1980-549720230005.supl.1)
Supplement: Supplementary file 1 [file 1980-5497-rbepid-26-suppl1-e230005-suppl1.pdf]

Material Suplementar - Violência contra pessoas LGB+ no Brasil: análise da Pesquisa Nacional de Saúde, 2019

Tabela 1. Distribuição dos adultos brasileiros por orientação sexual, com respectivos intervalos de confiança, segundo características sociodemográficas. Pesquisa Nacional de Saúde, 2019.

Variáveis	Total	Distribuição (Intervalo de Confiança 95%)			
		Heterossexual	LGB+	Não sabe	Recusou-se a responder
**Sexo**					
Masculino	100,00	94,70 (94,25 - 95,12)	1,93 (1,69 - 2,20)	1,08 (0,89 - 1,32)	2,29 (2,00 - 2,62)
Feminino	100,00	94,80 (94,43 - 95,16)	1,86 (1,64 - 2,11)	1,08 (0,92 - 1,26)	2,26 (2,02 - 2,52)
**Faixa etária**					
18 a 29 anos	100,00	89,79 (88,87 - 90,64)	4,91 (4,31 - 5,59)	2,11 (1,73 - 2,57)	3,19 (2,72 - 3,75)
30 a 39 anos	100,00	95,36 (94,84 - 95,83)	1,92 (1,60 - 2,30)	0,97 (0,77 - 1,22)	1,75 (1,48 - 2,08)
40 a 59 anos	100,00	96,00 (95,54 - 96,41)	0,99 (0,84 - 1,15)	0,80 (0,63 - 1,03)	2,21 (1,86 - 2,62)
60 ou mais	100,00	97,22 (96,82 - 97,56)	0,25 (0,19 - 0,34)	0,58 (0,42 - 0,80)	1,95 (1,66 - 2,29)
**Escolaridade**					
Sem instrução e fundamental incompleto	100,00	95,91 (95,49 - 96,29)	0,50 (0,39 - 0,64)	1,30 (1,08 - 1,57)	2,29 (2,00 - 2,62)
Fundamental completo e médio incompleto	100,00	92,73 (91,69 - 93,66)	2,56 (1,94 - 3,36)	1,63 (1,27 - 2,10)	3,08 (2,54 - 3,71)
Médio completo e superior incompleto	100,00	94,30 (93,80 - 94,76)	2,41 (2,11 - 2,74)	1,02 (0,81 - 1,28)	2,27 (1,96 - 2,63)

Superior	100,00	95,07 (94,38 - 95,68)	3,20 (2,76 - 3,72)	0,21 (0,11 - 0,37)	1,52 (1,15 - 2,01)
**Raça/cor da pele**					
Branca	100,00	95,25 (94,81 - 95,66)	1,83 (1,59 - 2,11)	0,81 (0,63 - 1,03)	2,11 (1,81 - 2,45)
Negra	100,00	94,35 (93,95 - 94,73)	1,93 (1,71 - 2,18)	1,30 (1,13 - 1,50)	2,42 (2,18 - 2,69)
Outras	100,00	95,26 (93,33 - 96,66)	2,24 (1,24 - 3,98)	0,69 (0,34 - 1,39)	1,81 (1,10 - 2,98)
**Região de residência**					
Norte	100,00	93,38 (92,45 - 94,20)	1,89 (1,50 - 2,38)	1,95 (1,49 - 2,55)	2,78 (2,32 - 3,33)
Nordeste	100,00	95,24 (94,79 - 95,65)	1,55 (1,34 - 1,81)	1,08 (0,91 - 1,28)	2,13 (1,84 - 2,46)
Sudeste	100,00	94,74 (94,18 - 95,25)	2,10 (1,79 - 2,46)	0,94 (0,71 - 1,25)	2,22 (1,88 - 2,63)
Sul	100,00	94,66 (94,02 - 95,24)	1,95 (1,61 - 2,35)	0,86 (0,66 - 1,13)	2,53 (2,11 - 3,02)
Centro-oeste	100,00	94,76 (94,06 - 95,38)	1,77 (1,46 - 2,14)	1,38 (1,02 - 1,87)	2,09 (1,69 - 2,58)
**Renda domiciliar**					
Até 01 SM	100,00	94,57 (94,15 - 94,95)	1,52 (1,33 - 1,74)	1,47 (1,27 - 1,70)	2,44 (2,20 - 2,72)
Mais de 01 a 03 SM	100,00	94,96 (94,44 - 95,43)	1,96 (1,68 - 2,30)	0,78 (0,60 - 1,01)	2,30 (1,97 - 2,69)
Acima de 03 SM	100,00	94,94 (94,13 - 95,64)	3,30 (2,73 - 3,99)	0,32 (0,19 - 0,55)	1,44 (1,09 - 1,91)
**Local de moradia**					
Urbano	100,00	94,74 (94,41 - 95,05)	2,05 (1,87 - 2,26)	1,01 (0,87 - 1,17)	2,20 (1,99 - 2,43)
Rural	100,00	94,84 (94,18 - 95,44)	0,88 (0,64 - 1,21)	1,52 (1,24 - 1,86)	2,76 (2,33 - 3,27)
**Estado Civil**					
Solteira	100,00	91,62 (91,05 - 92,15)	3,81 (3,45 - 4,20)	1,81 (1,55 - 2,12)	2,76 (2,47 - 3,09)
Casada	100,00	96,92 (96,54 - 97,26)	0,48 (0,38 - 0,61)	0,57 (0,45 - 0,72)	2,03 (1,73 - 2,38)
Viúva	100,00	97,56 (96,92 - 98,07)	0,18 (0,001 - 0,38)	0,49 (0,25 - 0,97)	1,77 (1,37 - 2,27)

Divorciada, desquitada ou separada judicialmente	100,00	97,31 (96,65 - 97,84)	0,88 (0,66 - 1,18)	0,43 (0,25 - 0,75)	1,38 (0,97 - 1,96)
**Total**	100,00	94,75 (94,46 - 95,03)	1,89 (1,73 - 2,07)	1,08 (0,95 - 1,23)	2,28 (2,08 - 2,48)

Fonte: Instituto Brasileiro de Geografia e Estatística
