# Supplementary material for: Violence Against LGB+ people in Brazil: analysis of the 2019 National Survey of Health
Source: Rev Bras Epidemiol. 2023 Apr 21;26(Suppl 1):e230005. doi: 10.1590/1980-549720230005.supl.1 (PMC10176725; doi:10.1590/1980-549720230005.supl.1)
Supplement: Translation

# Translation: Violência contra pessoas LGB+ no Brasil: análise da Pesquisa Nacional de Saúde 2019

**DOI:** 10.1590/1980-549720230005.supl.1.1

**Authors:** Nádia Machado de Vasconcelos, Francielle Thalita Almeida Alves, Gisele Nepomuceno de Andrade, Isabella Vitral Pinto, Adauto Martins Soares, Cimar Azeredo Pereira, Deborah Carvalho Malta

**Affiliations:** I Universidade Federal de Minas Gerais, Faculdade de Medicina, Programa de Pós-Graduação em Saúde Pública – Belo Horizonte (MG), Brasil.; II Universidade Federal de Minas Gerais, Faculdade de Medicina – Belo Horizonte (MG), Brasil.; III Universidade Federal de Minas Gerais, Faculdade de Medicina, Programa de Pós-Graduação em Enfermagem – Belo Horizonte (MG), Brasil.; IV Ministério da Saúde, Departamento de Doenças e Agravos Não Transmissíveis – Brasília (DF), Brasil.; V Instituto Brasileiro de Geografia e Estatística – Rio de Janeiro (RJ), Brasil.; VI Universidade Federal de Minas Gerais, Faculdade de Medicina, Departamento de Enfermagem Materno Infantil e Saúde Pública – Belo Horizonte (MG), Brasil.

**Keywords:** Minorias sexuais e de gênero, Discriminação sexual, Violência de gênero, Inquéritos epidemiológicos

## Abstract

**Objetivo:**

Analisar a associação entre a orientação sexual autoidentificada e a violência na população brasileira.

**Métodos:**

Estudo epidemiológico transversal que utilizou base de dados da Pesquisa Nacional de Saúde 2019. Analisaram-se a violência total e seus subtipos (psicológica, física e sexual) nos 12 meses anteriores. Estimou-se a prevalência e a odds ratio ajustada por faixa etária, com seus respectivos intervalos de confiança de 95%, segundo orientação sexual autoidentificada da população acima de 18 anos no Brasil. Considerou-se a significância estatística de 5%.

**Resultados:**

A população brasileira autoidentificou-se majoritariamente como heterossexual (94,75%), e 1,89% identificou-se como LGB+. Esse percentual foi inferior ao de entrevistados que se recusaram a responder à pergunta (2,28%). A prevalência da violência na população geral do Brasil foi de 18,27%, sendo o subtipo mais comum a violência psicológica (17,36%). A população LGB+ apresentou mais que o dobro de chances de sofrer qualquer tipo de violência. As mulheres LGB+ apresentaram as maiores prevalências de todos os subtipos de violência e os homens heterossexuais, as menores. Mulheres LGB+ tiveram mais de três vezes mais chances de sofrer violência física, comparadas às mulheres heterossexuais. Enquanto isso, homens LGB+ mostraram chances quase oito vezes maiores de sofrer violência sexual que os homens heterossexuais.

**Conclusão:**

A violência contra a população LGB+ apresentou alta prevalência no país. São necessárias políticas públicas voltadas a essa população para que se enfrente o preconceito contra a diversidade sexual e seja possível garantir os direitos das pessoas não heterossexuais.

## INTRODUÇÃO

A cis-heteronormatividade pode ser entendida como um sistema de relações de poder em que se pressupõe a existência de apenas dois gêneros opostos (homem e mulher), que são sempre coincidentes com os corpos (sexo biológico masculino e feminino) e que sempre se atrairão mutualmente por seu oposto^1^. No entanto, a orientação sexual e a identidade de gênero podem assumir múltiplas características e comportamentos afetivo-sexuais que diferem dessa normatividade. As pessoas que apresentam essa autoidentificação dita dissidente compõem a população LGBTQIA+, termo guarda-chuva que engloba, mas não se limita a, pessoas lésbicas, *gays*, bissexuais, transgêneros, *queer*, intersexo, assexuais e outras^2^. A população de lésbicas, *gays*, bissexuais e outras minorias sexuais (LGB+), objeto deste estudo, é composta de indivíduos que apresentam comportamentos, desejos e/ou identidade emotivo-afetivo-sexuais diferentes dos definidos para os heterossexuais cisgêneros^3,4^. Pelo fato de diferir do esperado enquanto padrão estabelecido, essa população encara discriminação, vulnerabilidades e invisibilidade, sofrendo o chamado preconceito contra a diversidade sexual^5^.

Uma das consequências desse preconceito é a maior susceptibilidade dessa população à violência. A violência é um problema de saúde pública, e seu enfrentamento faz parte dos Objetivos de Desenvolvimento Sustentável da Agenda 2030^6^. Pessoas expostas à violência podem apresentar desfechos desfavoráveis em saúde, tanto psicológicos como físicos e sexuais. Entre as principais consequências de atos violentos, podem-se destacar depressão, transtorno do estresse pós-traumático, fraturas, traumatismos cranianos, além de contaminação por infecções sexualmente transmissíveis e gravidez não desejada^7^.

Relatório do Grupo Gay da Bahia (GGB), organização não governamental (ONG) que desde 1980 recolhe dados sobre a violência contra as minorias sexuais e de gênero no Brasil, mostrou que 165 *gays* e lésbicas foram mortos no país apenas em 2021, em consequência do preconceito por suas orientações sexuais^8^. Além disso, estudo com dados do Sistema de Informação de Agravos de Notificação (SINAN) de 2015 a 2017 encontrou 13.129 notificações contra homossexuais e bissexuais, dado que pode estar subestimado em razão das possíveis subnotificações^9^. Para além disso, é necessário destacar que existe escassez de informações sobre esse grupo no Brasil, o que dificulta o conhecimento do perfil dessa população e o reconhecimento de suas necessidades, impactando a formulação de políticas públicas voltadas para essa minoria social^10^.

A fim de avançar nesse sentido, a Pesquisa Nacional de Saúde (PNS) de 2019 incluiu, pela primeira vez em inquérito epidemiológico nacional do Instituto Brasileiro de Geografia e Estatística (IBGE), a investigação sobre a orientação sexual de brasileiros adultos^11^. A inclusão dessa temática levanta múltiplas possibilidades de análise quanto à saúde e aos fatores de risco e proteção da população LGB+, entre elas a violência.

Salienta-se que o estudo sobre a violência tem grande relevância no contexto da população LGB+, uma vez que permite compreender a complexidade da vulnerabilidade a que esse grupo está exposto. Assim, conhecer as características desse agravo favorece a implementação e o fortalecimento de políticas públicas que visem ao enfrentamento do preconceito contra essa população.

Nessa perspectiva, pela primeira vez, o presente estudo teve como objetivo analisar a associação entre a orientação sexual autoidentificada e a violência na população brasileira.

## MÉTODO

### Delineamento e fonte de dados

Estudo epidemiológico transversal, de caráter analítico e abordagem quantitativa, que utilizou dados da PNS 2019.

A PNS 2019 avaliou indivíduos de 15 anos ou mais de idade residentes no Brasil. A amostra constituiu-se em um plano conglomerado de três estágios de seleção, a saber: 1. setores censitários ou conjunto de setores; 2. domicílios; e 3. moradores. Foram selecionados 108.525 domicílios para a entrevista, e a amostra final foi de 90.846 entrevistas realizadas, com taxa de resposta de 96,5%. Maiores detalhes sobre a metodologia da PNS 2019 podem ser vistos em publicação específica^12^. Para o presente estudo, selecionaram-se as pessoas com 18 anos ou mais que responderam ao módulo de violência e atividade sexual.

### Variáveis

A variável desfecho, violência, foi construída utilizando-se as perguntas do módulo de violência (V). Considerou-se a presença de violência quando o entrevistado respondeu “sim” para alguma das alternativas das perguntas do questionário referentes às violências psicológica, física e sexual (detalhes do questionário podem ser vistos em: https://www.pns.icict.fiocruz.br/wp-content/uploads/2021/02/Questionario-PNS-2019.pdf).

Já a orientação sexual, variável explicativa, foi considerada com base na resposta para a pergunta “Qual é sua orientação sexual?”, contida no módulo de Atividade Sexual (Y) e categorizada em heterossexual; LGB+ (homossexual, bissexual e outra orientação); não sabe; e recusou-se a responder.

Selecionaram-se ainda variáveis sociodemográficas descritivas: sexo (masculino e feminino), faixa etária (18 a 29 anos; 30 a 39 anos; 40 a 59 anos; 60 anos ou mais); escolaridade (sem instrução e fundamental incompleto; fundamental completo e médio incompleto; médio completo e superior incompleto; e superior); raça/cor da pele (branca, negra [preta e parda]; outras [amarela e indígena]); Região (Norte, Nordeste, Sudeste, Sul e Centro-Oeste); renda (até um salário-mínimo (SM); mais de um a três SM; acima de três SM); local de moradia (urbano ou rural); e estado civil (solteiro; casado; viúvo; divorciado, desquitado ou separado judicialmente).

### Análise de dados

Na análise descritiva, calcularam-se as distribuições e seus respectivos intervalos de confiança de 95% (IC95%) da orientação sexual, assim como sua distribuição segundo características sociodemográficas. Calcularam-se, ainda, a prevalência da violência total e seus subtipos, total e estratificada por sexo autodeclarado na PNS.

Para avaliar a associação da violência à orientação sexual, realizou-se análise multivariada entre as variáveis desfecho (violência total, violência psicológica, violência física e violência sexual) e cada categoria da variável explicativa (orientação sexual). Estudos anteriores^13,14^ já demonstraram que a faixa etária é importante fator associado à violência e, por isso, pode ser considerada um confundidor. Dessa forma, optou-se por estimar a *odds ratio* ajustada (ORaj) pela faixa etária e respectivos IC95%, utilizando-se a regressão logística, técnica adequada e validada para estudos transversais^15^. O modelo final foi considerado ao nível de significância de 5%.

Graças ao desenho amostral complexo e às probabilidades distintas de seleção, a análise da PNS lança mão de pesos amostrais, e o peso final utilizado é o produto do inverso das expressões de chance de seleção de cada estágio da amostra. Ressalta-se que o peso final compreende a correção de não respostas e ajustes dos totais populacionais^12^.

Para as análises do presente estudo, o *Software for Statistics and Data Science* (Stata), versão 14.0, foi utilizado em seu módulo *survey*, que considera efeitos do plano amostral.

### Aspectos éticos

Todos os participantes informaram consentimento no momento da entrevista. O projeto da PNS foi aprovado pela Comissão Nacional de Ética em Pesquisa/Conselho Nacional de Saúde sob o Parecer nº 3.529.376, emitido em 23 de agosto de 2019.

## RESULTADOS

Foram analisadas 88.531 pessoas de 18 anos ou mais que responderam ao módulo Violência e Atividade Sexual na PNS. Os entrevistados autoidentificaram-se majoritariamente como heterossexuais (94,75%; IC95% 94,46–95,03), e 1,89% (IC95% 1,73–2,07) autoidentificaram-se como LGB+. Esse percentual foi inferior ao de entrevistados que se recusaram a responder à pergunta (2,28%; IC95% 2,08–2,48) (Tabela 1).

**Tabela 1:** Distribuição da orientação sexual autoidentificada dos brasileiros de 18 anos ou mais (n=88.531). Pesquisa Nacional de Saúde, 2019.

Orientação sexual	Distribuição (IC95%)		
	Total	Homens	Mulheres
**Heterossexual**	**94,75 (94,46–95,03)**	**94,70 (94,25–95,12)**	**94,80 (94,43–95,16)**
**LGB+**	**1,89 (1,73–2,07)**	**1,93 (1,69–2,20)**	**1,86 (1,64–2,11)**
Bissexual	0,68 (0,58–0,80)	0,50 (0,38–0,66)	0,84 (0,69–1,03)
Homossexual	1,15 (1,03–1,29)	1,40 (1,21–1,62)	0,93 (0,79–1,11)
Outra orientação	0,06 (0,03–0,08)	0,03 (0,01–0,05)	0,09 (0,05–0,14)
**Não sabe**	**1,08 (0,95–1,23)**	**1,08 (0,89–1,32)**	**1,08 (0,92–1,26)**
**Recusou-se a responder**	**2,28 (2,08–2,48)**	**2,29 (2,00–2,62)**	**2,26 (2,02–2,52)**
**Total**	100,00	100,00	100,00

IC95%: intervalo de confiança de 95%.

Com relação às características sociodemográficas, entre as faixas etárias, as pessoas de 18 a 29 anos foram as com maior autoidentificação de orientação LGB+ (4,91%; IC95% 4,31–5,59) e maior recusa de resposta (3,19%; IC95% 2,72–3,75). Pessoas com renda domiciliar acima de três SM também tiveram maior percentual de autoidentificação LGB+ (3,30%; IC95% 2,73–3,99), assim como aquelas que moram em zona urbana (2,05%; IC95% 1,87–2,26). Quanto ao estado civil, pessoas solteiras tiveram maior percentual de autoidentificação LGB+ (3,81%; IC95% 3,45–4,20), assim como de recusa à resposta (2,76%; IC95% 2,47–3,09) (Material Suplementar — Tabela 1).

A prevalência da violência total no Brasil em 2019 foi de 18,27% (IC95% 17,74–18,81). Em todos os subtipos de violência, as maiores prevalências ocorreram entre mulheres LGB+ [(psicológica: 40,53%; IC95% 34,29–47,09); (física: 15,84%; IC95% 10,99–22,28); (sexual: 5,50%; IC95% 3,23–9,20)], e as menores prevalências foram entre homens heterossexuais [(psicológica: 15,33%; IC95% 14,62–16,08); (física: 3,71%; IC95% 3,39–4,06); (sexual: 0,34%; IC95% 0,25–0,46)] (Figura 1).

**Figura 1:** Prevalência, com intervalo de confiança de 95%, da violência e seus subtipos, estratificada por sexo e orientação sexual. Pesquisa Nacional de Saúde, 2019.

Com relação à violência total, as pessoas LGB+ tiveram 2,52 (p<0,001) mais chances de sofrer violência que as heterossexuais, e os homens que se declararam LGB+ tiveram 2,69 (p<0,001) mais chances de sofrer violência que os homens heterossexuais, e as mulheres LGB+ tiveram 2,40 (p<0,001) mais chances de sofrer violência que as heterossexuais. Percentuais semelhantes foram encontrados para a violência psicológica (Tabela 2).

**Tabela 2:** Prevalência e *odds ratio* ajustada, com intervalos de confiança de 95%, da violência total e seus subtipos sofridos por brasileiros de 18 anos ou mais, segundo orientação sexual e estratificados por sexo. Pesquisa Nacional de Saúde, 2019.

		Prevalência e odds ratio ajustada (intervalo de confiança 95%)					
		Total		Masculino		Feminino	
		Prevalência (IC95%)	*Odds ratio* * (IC95%)	Prevalência (IC95%)	*Odds ratio* * (IC95%)	Prevalência (IC95%)	*Odds ratio* * (IC95%)
**Violência total**							
**Total**		18,27 (17,74–18,81)	–	17,01 (16,28–17,77)	–	19,38 (18,70–20,08)	–
**Orientação sexual**							
	Heterossexual	17,61 (17,08–18,16)	1,00 (–)	16,35 (15,62–17,11)	1,00 (–)	18,73 (18,04–19,43)	1,00 (–)
	LGB+	41,63 (37,14–46,27)	**2,52** **(2,08–3,07)**	40,67 (34,46–47,19)	**2,69** **(2,05–3,53)**	42,51 (36,26–49,01)	**2,40** **(1,83–3,14)**
	Não sabe	29,27 (24,10–35,03)	**1,64** **(1,27–2,13)**	25,46 (18,23–34,37)	1,42 (0,93–2,16)	32,62 (25,80–40,26)	**1,84** **(1,33–2,54)**
	Recusou-se a responder	20,96 (17,60–24,77)	1,16 (0,94–1,44)	20,44 (15,21–26,91)	1,17 (0,82–1,67)	21,43 (17,42–26,07)	1,16 (0,89–1,51)
**Violência psicológica**							
**Total**		17,36 (16,84–17,90)	–	15,98 (15,26–16,72)	–	18,58 (17,91–19,27)	–
**Orientação sexual**							
	Heterossexual	16,73 (16,21–17,27)	1,00 (–)	15,33 (14,62–16,08)	1,00 (–)	17,96 (17,29–18,65)	1,00 (–)
	LGB+	40,03 (35,53–44,71)	**2,54** **(2,08–3,10)**	39,48 (33,26–46,06)	**2,79** **(2,12–3,67)**	40,53 (34,29–47,09)	**2,35** **(1,78–3,09)**
	Não sabe	26,64 (21,56–32,42)	**1,54** **(1,17–2,03)**	22,41 (15,29–31,60)	1,31 (0,83–2,07)	30,38 (23,56–38,19)	**1,75** **(1,24–2,46)**
	Recusou-se a responder	20,42 (17,08–24,21)	1,20 (0,97–1,50)	19,70 (14,53–26,14)	1,22 (0,85–1,74)	21,06 (17,06–25,70)	1,19 (0,91–1,56)
**Violência física**							
**Total**		4,15 (3,89–4,42)	–	4,05 (3,71–4,41)	–	4,24 (3,84–4,67)	–
**Orientação sexual**							
	Heterossexual	3,82 (3,57–4,09)	1,00 (–)	3,71 (3,39–4,06)	1,00 (–)	3,92 (3,54–4,34)	1,00 (–)
	LGB+	14,70 (11,16–19,12)	**3,00** **(2,19–4,12)**	13,45 (8,74–20,13)	**2,84** **(1,74–4,65)**	15,84 (10,99–22,28)	**3,18** **(2,09–4,83)**
	Não sabe	12,35 (8,33–17,92)	**2,84** **(1,84–4,38)**	13,93 (8,01–23,13)	**3,18** **(1,72–5,88)**	10,95 (6,21–18,59)	**2,52** **(1,36–4,67)**
	Recusou-se a responder	4,94 (3,52–6,88)	1,19 (0,82–1,71)	5,37 (3,32–8,59)	1,25 (0,74–2,10)	4,55 (2,81–7,26)	1,13 (0,68–1,88)
**Violência sexual**							
**Total**		0,76 (0,65–0,90)	–	0,45 (0,35–0,57)	–	1,05 (0,85–1,28)	–
**Orientação sexual**							
	Heterossexual	0,68 (0,56–0,82)	1,00 (–)	0,34 (0,25–0,46)	1,00 (–)	0,97 (0,78–1,22)	1,00 (–)
	LGB+	4,86 (3,21–7,28)	**4,98** **(3,10–7,99)**	4,15 (2,15–7,86)	**7,76** **(3,38–17,81)**	5,50 (3,23–9,20)	**3,95** **(2,21–7,05)**
	Não sabe	1,71 (0,94–3,11)	**1,96** **(1,03–3,72)**	2,15 (0,92–4,92)	**4,29** **(1,68–10,94)**	1,33 (0,60–2,90)	1,11 (0,48–2,57)
	Recusou-se a responder	0,56 (0,31–1,02)	0,73 (0,39–1,36)	0,79 (0,36–1,72)	1,87 (0,79–4,42)	0,35 (0,15–0,85)	**0,34** **(0,13–0,85)**

Nota:

* *odds ratio* ajustada por faixa etária; valores em negrito — p<0,05; IC95%: intervalo de confiança de 95%.

No tocante à violência física, homens LGB+ tiveram 2,84 mais chances (p<0,001) que homens heterossexuais, e mulheres LGB+ apresentaram 3,18 mais chances (p<0,001) em relação às mulheres heterossexuais (Tabela 2).

Finalmente, as chances de uma pessoa LGB+ sofrer violência sexual foram 4,98 (p<0,001) maiores que as das pessoas heterossexuais. Homens LGB+ apresentaram 7,76 (p<0,001) mais chances de sofrer esse subtipo de violência que os homens heterossexuais, enquanto mulheres LGB+ tiveram 3,95 (p<0,001) mais chances de sofrê-la comparadas com as mulheres heterossexuais (Tabela 2).

## DISCUSSÃO

Esse estudo analisou dados da PNS 2019 e mostrou que aproximadamente 2% da população brasileira se autoidentifica como LGB+, percentual menor que o daqueles que se recusaram a responder à pergunta. Aproximadamente metade das pessoas LGB+ relataram ter sofrido algum tipo de violência nos últimos 12 meses, e essas pessoas tiveram aproximadamente o dobro de chances de sofrer qualquer subtipo de violência em relação às pessoas que se autoidentificaram como heterossexuais. As mulheres LGB+ foram as vítimas mais frequentes, enquanto os homens heterossexuais os com menor percentual. A razão de chances é mais alta para a violência sexual, e pessoas LGB+ mostraram chances quase cinco vezes maiores de serem vítimas desse subtipo de violência.

O percentual da população acima de 18 anos que se autoidentificou com LGB+ neste estudo ficou abaixo do encontrado por pesquisa da DataFolha realizada em 2018, que chegou ao percentual de 4,42% da população brasileira^16^. O percentual de pessoas que se recusaram a responder à pergunta sobre orientação sexual superou o de pessoas que se autoidentificaram como LGB+. Esse fato demonstra que a questão da diversidade sexual é estigmatizante no Brasil. A população LGB+ sofre historicamente com preconceitos e discriminações de cunho religioso, moral e até mesmo de assistência à saúde^17^. Dessa forma, muitas vezes as pessoas tentam esconder sua orientação dissidente, e a recusa a responder à questão pode ser uma forma de se proteger^10^.

Além disso, o percentual de pessoas que não souberam responder à questão foi semelhante ao de pessoas que se autoidentificaram como LGB+. Uma vez que a temática da orientação sexual foi abordada em uma única questão, pode-se imaginar que tenha havido dificuldade de entendimento da pergunta por parte dos entrevistados. Uma forma de mitigar esse fato seria incluir termos mais conhecidos, como lésbica e *gay* ^10^.

Este estudo mostrou que as pessoas LGB+ têm mais que o dobro de chances de sofrer qualquer violência, em todos os seus subtipos. O preconceito contra a diversidade sexual tem sua base na violência de gênero. O gênero pode ser entendido como uma construção social em que o sexo biológico ditaria o papel que uma pessoa deve exercer^18^. Nessa estrutura existe também a heteronormatividade, em que se exige das pessoas um estilo de vida padronizado, apresentando-se posturas e escolhas coerentes com o que se determinou como correto para homens e mulheres^19^. Assim, essa construção social não só impõe uma forma de agir e se portar publicamente, como também determina que cada um deve se relacionar afetiva e sexualmente com o gênero oposto^5^. Qualquer diferença desse padrão é então motivo de preconceito e justificativa para atos violentos^17^.

O presente estudo demonstrou que mulheres que se autoidentificaram como LGB+ são as que apresentaram maiores prevalências de violência de todos os subtipos, enquanto homens autoidentificados como heterossexuais foram os com menores prevalências. Esse achado demonstra que existe um aumento da vivência de violência com o acúmulo de vulnerabilidades sociais que as pessoas experienciam. Mulheres lésbicas e bissexuais sofrem com uma dupla discriminação: sexismo e preconceito contra a diversidade sexual. Em nossa sociedade, existe uma depreciação das mulheres diante da suposta supremacia masculina, além de uma desqualificação dos LGB+ baseada na normatização cis-heterossexista^20^. Dessa forma, mulheres lésbicas e bissexuais tornam-se mais sujeitas à violência, e uma revisão sistemática que analisou artigos com dados de 50 países encontrou prevalência de até 25% de violência física e de 13,2% de violência sexual nessa população^21^.

Pessoas que se autoidentificaram como LGB+ tiveram três vezes mais chances de sofrer violência física que as heterossexuais, sendo essas chances maiores na comparação entre as mulheres do que entre os homens. Esse achado é compatível com outro estudo que analisou as notificações de violência entre 2015 e 2017 e observou que, entre adultos, o principal subtipo de violência notificada é a física, e as lésbicas são as principais vítimas^9^. Mais uma vez, esse dado reforça a interseccionalidade de vulnerabilidades.

Este estudo verificou, ainda, que pessoas LGB+ apresentaram quase cinco vezes mais chances de sofrer violência sexual que pessoas heterossexuais, sendo este o subtipo de violência com maior diferença entre heterossexuais e LGB+. Entre muitas questões que envolvem essa situação, uma é a ocorrência de “estupro corretivo”, em que pessoas não heterossexuais sofrem abusos em que a intencionalidade do agressor é controlar o comportamento social e/ou sexual da vítima^22^.

Além disso, o estudo mostrou que os homens LGB+ tiveram aproximadamente oito vezes mais chances de sofrer tal violência em relação aos homens heterossexuais, enquanto as mulheres LGB+ tiveram perto de quatro vezes mais chances em comparação às mulheres heterossexuais. O estudo do DataFolha encontrou informação semelhante: a proporção de homens *gays* que relataram violência sexual foi mais de dez vezes maior que a de homens heterossexuais, enquanto mulheres lésbicas relataram aproximadamente 1,5 vez mais violência sexual que as mulheres heterossexuais^16^. Importante lembrar que os homens heterossexuais são os que menos sofreram esse tipo de violência e, em razão da baixa prevalência dessa violência e pequena amostra, essas porcentagens podem não ser fidedignas. Todavia, é necessário também considerar que os homens podem ter atos violentos para com seus parceiros por homofobia internalizada^23^ e criação social de resolução de conflitos por via violenta^24^. Mais estudos são necessários para entender melhor esse resultado.

Por comporem uma população vulnerável, as pessoas LGB+ demandam políticas públicas que garantam seus direitos humanos e à saúde. No Brasil, a atual constituição não versa especificamente sobre essa população, uma derrota para o movimento organizado pelos direitos LGBTQIA+ no país^25^. Entretanto, algumas conquistas foram feitas ao longo das últimas décadas, como a garantia do direito ao casamento civil entre pessoas do mesmo sexo em 2013 e a tipificação de atos preconceituosos como crime equiparado ao racismo, em 2019. Na área da saúde, foi desenvolvida em 2013 a Política Nacional de Saúde Integral de Lésbicas, *Gays*, Bissexuais, Travestis e Transexuais^26^ e, mais recentemente, houve o reconhecimento da possibilidade de pessoas homossexuais e bissexuais serem doadoras de sangue, em 2020^27^.

Nos últimos anos, porém, o Brasil vem passando por um período de crise sociopolítica, e o avanço do conservadorismo tem limitado as conquistas LGBTQIA+ e imposto retrocessos a essa população. Desde 2018, organismos voltados à proteção da população LGB+ foram extintos, como a Secretaria de Educação Continuada, Alfabetização, Diversidade e Inclusão e o Conselho Nacional de Combate à Discriminação LGBT, além de representantes do governo sistematicamente proferirem discursos de discriminação e ódio a essa população, até mesmo com representação legal para que se revogue a decisão de equiparação de homofobia ao racismo^28^. Dessa forma, as políticas públicas de proteção da população LGB+ ainda têm muito a avançar, com a construção de arcabouço jurídico que resguarde os direitos dessa população, previsão orçamentária para os planos e programas e maior representatividade política de pessoas LGB+^25^.

Os dados deste estudo referem-se ao último ano antes da pandemia de COVID-19 decretada em 2020 pela Organização Mundial da Saúde. No contexto da pandemia, o isolamento social, o aumento do estresse e a possível exposição a membros familiares desrespeitosos agravaram o risco de violência para a população LGB+^29^. Além disso, a insegurança econômica, com o aumento do desemprego e da pobreza no país, intensificou as fragilidades já enfrentadas por essas pessoas durante a crise pandêmica^30^. Dessa forma, os dados da PNS 2019 podem servir como linha de base para novas análises sobre a prevalência da violência contra pessoas LGB+ durante e após a crise sanitária mundial.

A PNS 2019 foi o primeiro inquérito epidemiológico domiciliar de base populacional brasileiro a trazer uma questão relacionada à orientação sexual, o que pode auxiliar nos estudos sobre essa população e produzir evidências científicas que auxiliem na construção de políticas públicas eficazes para a garantia de direitos dessas pessoas. Apesar disso, ainda é necessário ampliar o questionário, incluindo perguntas relacionadas à identidade de gênero, de forma a trazer visibilidade a todas as vulnerabilidades a que pessoas não heterocisnormativas estão expostas.

Entre as limitações deste estudo, destacamos que os dados sobre orientação sexual são considerados experimentais, e o IBGE chama a atenção para o fato de que as análises devem ser feitas com cautela. Além disso, a amostra não contempla populações de rua, asilos, quilombos e aldeias, além de o inquérito não incluir todos os subtipos de violência, deixando de fora a violência moral e patrimonial, por exemplo. Ademais, pode-se supor que haja subestimação das prevalências aqui calculadas, dado o estigma que a violência e a orientação não heterossexual têm na sociedade, levando as pessoas a não relatar esses fatos. Para tentar minimizar esta última limitação, poder-se-ia garantir a privacidade dos entrevistados no momento de resposta aos módulos referentes a tais perguntas, além de permitir resposta direta no dispositivo, diminuindo o possível constrangimento.

Em conclusão, os dados da PNS 2019 demonstram alta prevalência de violência contra pessoas LGB+ no Brasil, destacando que essa população apresenta grande vulnerabilidade para tal agravo. Dessa forma, este estudo contribui com evidências científicas da necessidade de uma articulação intersetorial que envolva saúde, educação, justiça, segurança pública, entre outros, no enfrentamento dessa violência, com a criação de políticas públicas que ajam de forma integral na proteção da vida e dos direitos das pessoas LGB+.
